# Spatial Decomposition of a Broadband Pulse Caused by Strong Frequency Dispersion of Sound in Acoustic Metamaterial Superlattice

**DOI:** 10.3390/ma14010125

**Published:** 2020-12-30

**Authors:** Yuqi Jin, Yurii Zubov, Teng Yang, Tae-Youl Choi, Arkadii Krokhin, Arup Neogi

**Affiliations:** 1 Department of Physics, University of North Texas, P.O. Box 311427, Denton, TX 76203, USA; yuqijin@my.unt.edu (Y.J.); yuriizubov@my.unt.edu (Y.Z.); tengyang@my.unt.edu (T.Y.); arkadii.krokhin@unt.edu (A.K.); 2 Department of Mechanical Engineering, University of North Texas, 3940 North Elm Suite, Denton, TX 76207, USA; tae-youl.choi@unt.edu; 3 Department of Materials Science and Engineering, University of North Texas, 3940 North Elm Suite, Denton, TX 76207, USA; 4 Center for Agile and Adaptive Additive Manufacturing, 3940 North Elm Suite, Denton, TX 76207, USA

**Keywords:** acoustic metamaterial, ultrasound pulse, pulse decomposition, acoustic superlattice

## Abstract

An acoustic metamaterial superlattice is used for the spatial and spectral deconvolution of a broadband acoustic pulse into narrowband signals with different central frequencies. The operating frequency range is located on the second transmission band of the superlattice. The decomposition of the broadband pulse was achieved by the frequency-dependent refraction angle in the superlattice. The refracted angle within the acoustic superlattice was larger at higher operating frequency and verified by numerical calculated and experimental mapped sound fields between the layers. The spatial dispersion and the spectral decomposition of a broadband pulse were studied using lateral position-dependent frequency spectra experimentally with and without the superlattice structure along the direction of the propagating acoustic wave. In the absence of the superlattice, the acoustic propagation was influenced by the usual divergence of the beam, and the frequency spectrum was unaffected. The decomposition of the broadband wave in the superlattice’s presence was measured by two-dimensional spatial mapping of the acoustic spectra along the superlattice’s in-plane direction to characterize the propagation of the beam through the crystal. About 80% of the frequency range of the second transmission band showed exceptional performance on decomposition.

## 1. Introduction

Artificial phononic periodic structures have been studied for decades and are generally termed phononic crystals [1]. A phononic crystal can manipulate the propagation of a wave based on the structure of the crystal. It depends on the frequency and direction of the incident wave with respect to the crystal plane, which can be very different from natural crystals [2]. In the long-wavelength limit, the phononic crystals’ physical properties can be artificially designed according to the effective medium theory [3,4,5]. Once the operating wavelength approaches the periodicity or smaller, the eigenmodes’ dispersion relation becomes highly nonlinear and may exhibit anomalous group velocity [6]. In this region, the phononic crystals behave as metamaterials [7,8,9,10]. The potential wave steering functionalities of elastic [11], acoustic [12], and thermal waves [13] using 1D, 2D, or 3D phononic periodic structures have been demonstrated along with the fundamental principles in the existing studies. In those transmission bands, the abnormal behavior includes negative refraction [14] and flat regions of the equifrequency surface [9,15]. These unique properties provide opportunities to realize long-distance acoustic collimator [8] and super-resolution monostatic [16,17] and bistatic [18,19,20] lenses, which showed great improvement in acoustic detection [21] and ultrasonic imaging [22,23,24] and elastography [25,26,27].

The acoustic superlattice structure considered in this work is a 1D layered and periodic structure, which possesses most of the properties of metamaterials [28]. These structures are commonly designed to modify the propagation of thermal phonons in thermo-electrical devices [29]. A recent study shows that the introduction of randomness in the periodicity of a superlattice structure leads to Anderson localization of thermal phonons [30], leading to higher efficiency than conventional designs. At the macroscope scale, solid-solid periodic superlattices are used to steer a propagating Rayleigh wave and realize filtering [31] and waveguiding [32].

This study proposes a numerical and experimental realization of the spectral and spatial deconvolution of an acoustic broadband pulse into frequency components using an acoustic metamaterial superlattice. The superlattice design was introduced in our previous work on non-spreading propagation of acoustic beam [8]. Here, we focus on the decomposition behavior of the superlattice. The decomposition of the signal into multi-spectral components occurs due to the frequency-dependent index of refraction and frequency-dependent refraction angle. This rainbow effect originates from frequency dispersion and is well-known in optics. Here we demonstrate that it can be strongly enhanced for sound waves due to strong frequency dispersion of the eigenmodes at the second transmission band. With a broadband pulse source incident on the superlattice’s facet, the monochromatic components with different wavelengths propagate through the superlattice with self-sorted refraction paths. The frequency components from short to long wavelength are transmitted with different refraction angles from large to small.

## 2. Materials and Methods

### 2.1. Design of the Superlattice Structure

**Figure 1:**
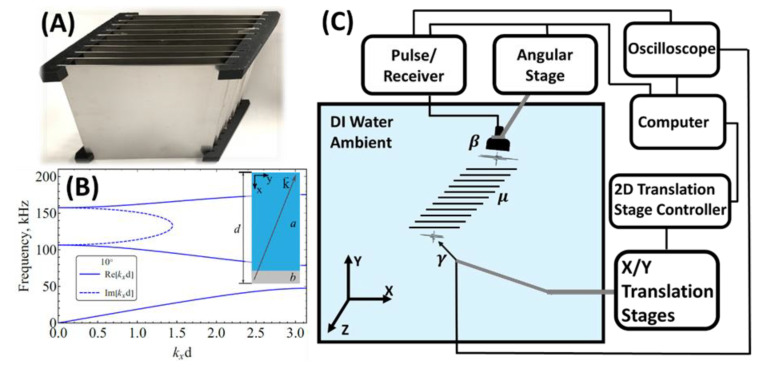
Design of the 10 layers superlattice structure. (**A**) Photograph of the real fabricated superlattice structure. The corner holders were made by water-property-like resin. (**B**) The band structure calculated for 10° angle of incidence. (**C**) Schematic diagram of the experimental setup. In the figure, β refers to the single element planar transducer with 100 kHz fundamental frequency, μ indicates the 10-layer superlattice structure, and γ is the needle type underwater hydrophone.

The periodic lattice structure was assembled from 10 pieces of 178 mm wide square shape stainless steel 409 plates with a thickness of 0.891 mm. The plates are arranged periodically with 10 mm period. The plates are held by stereolithography type Formlabs (Somerville, MA, USA) Form 2 printed sound transparent resin corner holders, as shown in Figure 1A. The superlattice is submerged in water. The scanned, transmitted area was 40 mm apart from the last stainless steel plate. The scanned line was 90 mm long with 1 mm interval.

Figure 1B shows the superlattice’s band structure calculated for the angle of incidence of 10° measured from axis *x*. The geometrical parameters of the unit cell (inset) are thickness of water (steel) layer *a =* 9.109 mm (*b* = 0.891 mm) and period *d =* 10 mm. The frequency bandwidth of the source pulse in this study was from 50 to 150 kHz. A pulse excites the eigenmodes in the second transmission band, which extends from 79 to 108 kHz. The bandgaps cut the harmonics with frequencies below 79 kHz and above 108 kHz.

### 2.2. Numerical Simulation

The numerical simulations were performed using finite element analysis (FEA) COMSOL Multiphysics 5.5 (Burlington, MA, USA) software. The experimental setup was modeled by placing the superlattice in the center of the 250 × 450 mm^2^ water tank. The parameters of DI water are: the speed of sound is 1480 m/s, and the density is 1000 kg/m^3^. The Young’s modulus, speed of sound, and density of the stainless steel plates are 200 GPa, 5750 m/s, and 7800 kg/m^3^, respectively. The transducer has a diameter of 1 inch (25.4 mm, the same size as the real transducer in the experiment). It is located 25 mm under the water level at the center of the left horizontal boundary. The distribution of pressure was calculated in the frequency domain for the angle of incidence of 10°.

### 2.3. Experimental Setup

Figure 1C illustrates a monostatic setup of the experiment. The superlattice is emerged in a large (550 × 550 × 550 mm^3^) DI water tank. Unfocused Ultran (State College, PA, USA) 25.4 mm diameter planar transducer NCG100-D25 generates 100 kHz fundamental frequency broadband pulse (from 50 to 200 kHz) in every 10 milliseconds, and the signal is measured by Müller-Platte (Oberursel, Germany) 1 mm diameter tip needle hydrophone. Pulse signal to transducer was generated by Imaginant (Pittsford, NY, USA) JSR 300 pulser/receiver. The measured time-domain signal from needle hydrophone was forwarded to Tektronix MDO 3024 (Beaverton, OR, USA) oscilloscope for further acquiring. The transducer was held and rotated in X-Y plane by a Thorlabs (Newton, NJ, USA) CR1-Z6 angular translation stage for variable incident angles. The needle hydrophone was held and moved in X-Y plane by a two-dimensional 200 by 200 mm translation range Newmark (Zimmerman, MN, USA) LC 200 linear translation stage, which was controlled by Newmark (Zimmerman, MN, USA) NSG-G2-X2 stepper motor controller. Before each experiment, the angular stage turned the transducer to 10° incident angle. The automatic raster scan was completed by a pre-prepared MathWorks (Natick, MA, USA) MATLAB R2020b code, which moved the needle hydrophone to each location and paused for 20 s for data acquirement. After recording each time window, the code moved the hydrophone to the next position.

## 3. Results

**Figure 2:**
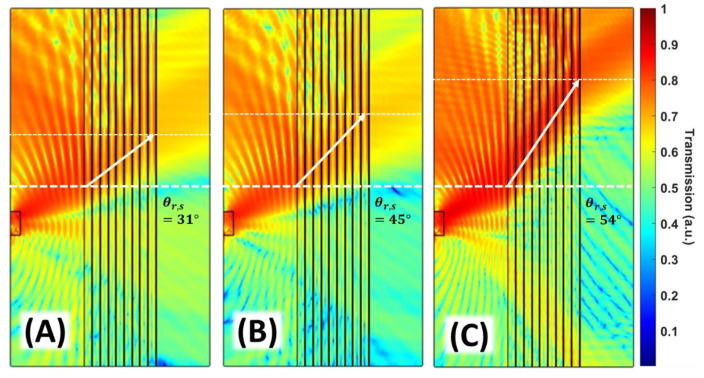
Numerical simulation of refraction of monochromatic beams incident to the 10 layers superlattice structure at 10° angle. Different refraction angles are observed for (**A**) 95 kHz, (**B**) 105 kHz, and (**C**) 115 kHz. The acoustic pressure distribution maps showed in normalized linear scale. $\theta_{r}$ refers to refraction angle. Subindex *s* was indicated simulation results.

The nature of the physical decomposition of the acoustic pulse by superlattice is the same as splitting white light by a spectrum of different colors when passing through a prism, namely the refraction coefficient’s frequency dispersion. Here we explore the frequency dispersion of sound at the second transmission band of the designed superlattice. Figure 2 demonstrates different refraction angles for monochromatic sound beams at three different frequencies that were incident on the superlattice at the same angle of incidence. On passing through 10 periods of the superlattice, the beams emerge out of the crystal at different spatial positions. The angle of refraction gradually increases with frequency. For frequencies 95, 105, and 115 kHz, we respectively obtain $n_{g}$ = 0.33, 0.24, and 0.21.

**Figure 3:**
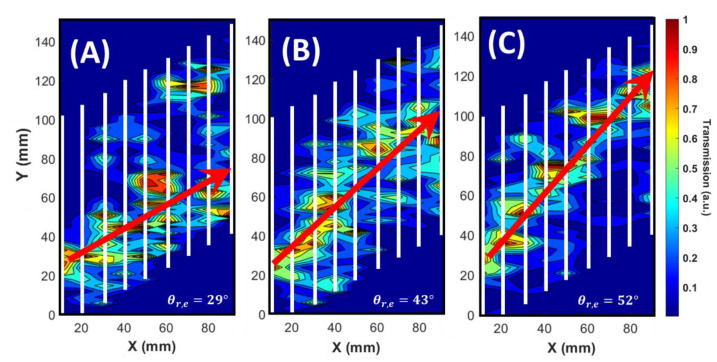
Experimental results of broadband pulse sourced sound intensity maps between the superlattice layers with 10° incidence to the structure. The subfigures illustrate the single frequency dependent refraction angles inside the superlattice obtained from Fourier transformation of the recorded time domain data. The acoustic intensity distribution maps are shown in normalized linear scale. (**A**) 95 kHz. (**B**) 105 kHz. (**C**) 115 kHz. $\theta_{r}$ refers to refraction angle. Subindex *e* indicates experimental results.

Figure 3 shows the experimental results for monochromatic beam refraction for the same frequencies used in numerical simulation in Figure 2. The pressure map for each frequency is obtained by Fourier transformation of experimentally recorded time-dependent wave envelopes. The time-domain data were measured at each middle point between the layers. The one-dimensional raster scan is formed from nine lines for constructing the contour plots in Figure 3. The incident beam hits the first layer near the point with coordinates (0, 25) on X-Y plane. The centers of the outcoming refracted beams with frequencies 95 kHz, 105 kHz, and 115 kHz were detected, respectively, around the points (90, 70), (90, 90), and (90, 110). Experimentally observed angles of refraction in Figure 3 are in good agreement with the numerical results in Figure 2.

**Figure 4:**
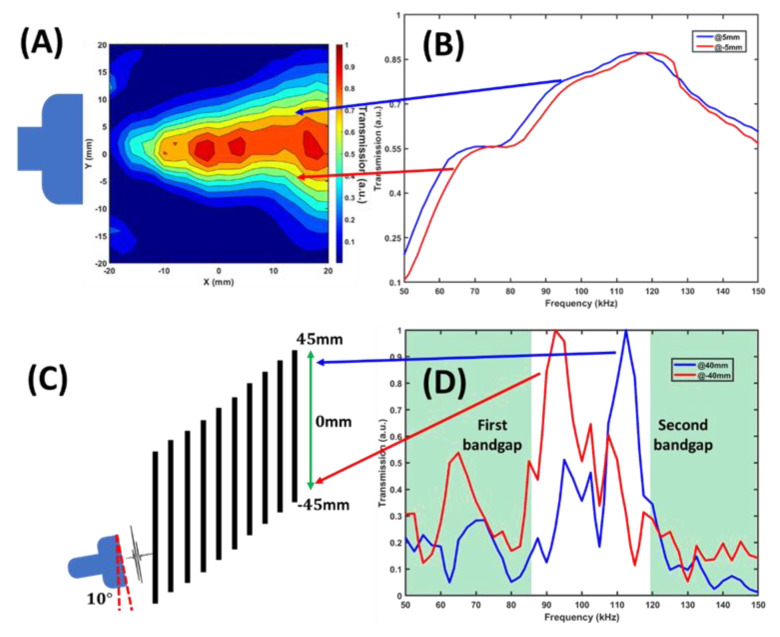
Experimental results of pulse decomposition measured at two symmetrical vertical points. (**A**) and (**B**) show the symmetric pressure map and almost equal transmission spectra obtained in free water without superlattice. (**C**) and (**D**) show different spectral compositions of the signals measured 40 mm above and below superlattice axis. The two spectra were measured at the distance 6 mm behind the last layer of the superlattice.

Experimental results obtained for the broadband pulse are presented in Figure 4. The transmission spectra were measured at two symmetric points behind the superlattice. For reference, the same measurements were performed without the superlattice, and the resultant pressure maps are shown in Figure 4A. The acoustic beam spreads symmetrically with respect to the horizontal axis, giving rise to two practically equal transmission spectra in Figure 4B. Unlike this, the spectra obtained at two symmetrical points 40 mm above and below the superlattice axis (see Figure 4C) are quite different. They are shown by red and blue curves in Figure 4D. Because of frequency dispersion inside the superlattice, the broadband pulse harmonics arrive at different vertical positions. Therefore, the spectral compositions of the signals measured above and below the superlattice axis are quite different, as shown in Figure 4D. The measured spectra exhibit narrow peaks at 92 and 112 kHz. Geometrical positions of these peaks coincide well with the positions calculated from Snell’s Law using the definition of the group index of refraction [33].

(1) $$n_{g}\left(\omega \right)=-\frac{k_{y}c_{0}/\omega }{{\left(\partial k_{x}/\partial k_{y}\right)}_{\omega }}\sqrt{1+{\left(\frac{\partial k_{x}}{\partial k_{y}}\right)}_{\omega }^{2}\;}$$

where $k_{x}$ is the Bloch vector of propagating wave, $k_{y}$ is the parallel to the plates component of the wave vector, which is conserved at refraction, and $c_{0}$ is the speed of sound in water. The derivative ${\left(\partial k_{x}/\partial k_{y}\right)}_{\omega }$ is calculated from the dispersion of the second transmission band at a given frequency ω.

**Figure 5:**
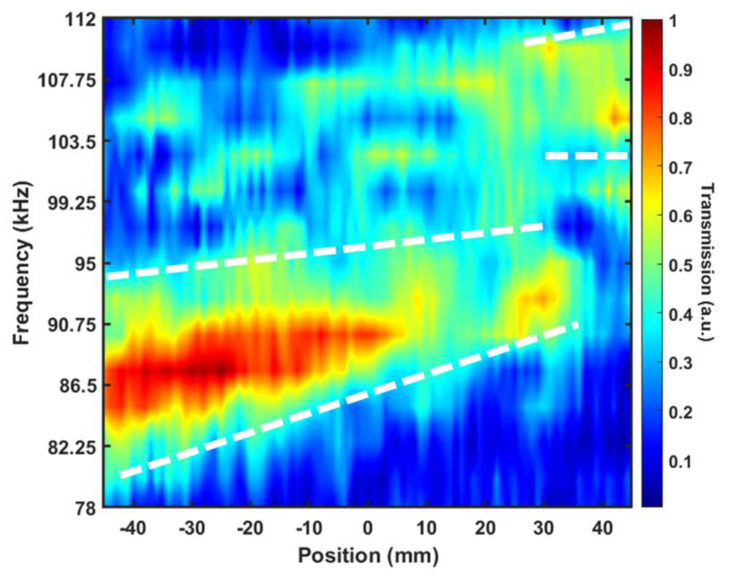
Experimental result of superlattice decomposition performance of the entire second transmission band along the scanned line at 2 mm parallel to the last layer of the superlattice. At different locations along the vertical axis, transmission peaks centered at different frequencies are observed. The 10-layer superlattice decomposes the pulse into narrow bands propagated along different refraction paths that finally arrive at different locations on the scanned line.

Continuous spatial distribution of pulse harmonics is given by color map in Figure 5. The transmitted intensity of sound was measured along the vertical 90 mm long vertical line (from −45 to +45 mm) parallel to the last plate at a distance 2 mm behind it. White dashed lines mark the region’s boundaries where the essential part of acoustic energy is transmitted. Inclinations of these lines are a direct manifestation of pulse decomposition in a medium with frequency dispersion.

The harmonics with frequencies from 82.25 to 95 kHz arrive within the spatial interval from −45 to 30 mm. The frequency harmonics from 100 to 110 kHz are refracted within the interval from 30 to 45 mm. The decomposition effect turns out to be relatively poor, since a broad transmission band from 88 to 108 kHz is observed between 21 to 36 mm. The decomposition performance is much better for the lower frequency range from 82.25 to 94.5 kHz. The decomposition performance at higher frequencies, between 102 to 110 kHz, is also rather effective. These harmonics are located from 37 to 45 mm on the vertical axis.

## 4. Discussion

Fundamental studies of periodic structures of acoustic metamaterials have been of high interest for decades. However, most of the practical applications of acoustic metamaterials have been mostly used for filtering, waveguiding, and lensing. This study demonstrates that an acoustic metamaterial superlattice can spatially disperse a broadband acoustic pulse into narrowband signals with different central frequencies. Due to refraction, the various components of the broadband wave travel with different velocities within the crystal and arrive at different locations at the end of the superlattice. To the best of our knowledge, a mechanical device has yet to be used for the spectral decomposition of a broadband pulse. Signal decomposition is conventionally done applying signal processing software and noise cancelation methods. Here we propose an alternative pure mechanical method, which is simple, robust, free from time delay, and does not require sophisticated numerical technique.

The decomposition effect is observed for the frequencies where the corresponding transmission band exhibits strongly nonlinear frequency dispersion. The proposed acoustic device’s performance can be increased if the sound wave propagates a long distance inside the superlattice and selects the interval of frequencies where the dispersion of the transmitting bands is stronger.

The intensity of the input acoustic beam is strongly reduced passing through 10 layers of the superlattice. The principal source of the weakness of the output signal is reflection at the water–superlattice interface. By estimating the maximum intensity of the beam obtained in numerical simulations, we conclude that average over the spectrum loss is about 11.8 dB. Experimentally, the loss averaged over all the frequency components is 22.9 dB. Higher experimental losses are due to viscous dissipation, which is ignored in numerical simulation and radiative losses to water environment generated by vibrating plate holders.

## Data Availability

Data available from corresponding author.
